# Sex Determination Using Frontal Sinus Diameters on Direct Radiography

**DOI:** 10.7759/cureus.47476

**Published:** 2023-10-22

**Authors:** Emre Emekli

**Affiliations:** 1 Department of Radiology, Etimesgut Şehit Sait Ertürk State Hospital, Ankara, TUR

**Keywords:** sex determination, skull radiography, direct radiography, frontal sinus, paranasal sinus

## Abstract

Background and aim: The shape and developmental stages of the frontal sinus (FS) differ in each individual, it can be used to determine sex and identity. This study aimed to assess the morphological structure of the FS diameters in sex determination using posterioanterior (AP) skull radiography. This data can be valuable for identifying and characterizing human features.

Methods: The study included 350 patients (171 females, 179 males) aged between 20 and 55 years who underwent AP skull radiography. FSs were classified as symmetrical, asymmetrical, unilateral, or bilateral aplasic. The right and left FS height and width measurements were made based on the largest diameters.

Results: The mean age was 32.8±11.45 years for the whole sample. The mean age of the males was 31.23±11.9 (20-51) years and that of the females was 34.45±10.75 (20-55) years. When classified anatomically, 20 patients had bilateral aplasia (12 males, eight females), 19 patients had right aplasia (10 males, nine females), and 11 patients had left aplasia (seven males, four females). FS was symmetrical in 206 (72.1%) patients while right dominance was observed in 33 (9.43%) patients and left dominance in 61 (17.43%) patients. The FS height and width values on both sides ​​were statistically significantly higher in males than in females (p<0.001 for each parameter). The females were accurately classified at a rate of 71.2% and the males at 68.2%.

Conclusion: Notably, our research has revealed that frontal sinus diameters are consistently larger in males than in females, and that direct radiography can be employed for gender determination with an accuracy rate of 69.7%. This information underscores the utility of morphometric evaluation of the frontal sinus diameters on direct radiography for gender and identity determination, potentially in conjunction with other parameters. In conclusion, our study has demonstrated the potential of the morphological structure of the frontal sinuses as a valuable tool for identifying and characterizing human individuals.

## Introduction

It may be necessary to identify skeletons that cannot be recognized for any reason [1]. In this context, sex determination plays an important role as the first step [2]. In cases where descriptive characteristics, such as DNA and fingerprints cannot be used, radiological identification has an important place. Forensic anthropologists utilize various anatomical regions for sex determination in unidentified skeletons [3,4]. Paranasal sinuses are among the anatomical regions that can be used for sex determination. The frontal sinus (FS) is absent at the beginning of life, unlike other sinuses [5]. FS begins to develop as pea-shaped pockets located on both sides of the orbit. These pockets, which start to develop at the age of two, can only be detected radiologically when individuals reach the age of six [5]. FS completes its development by 20 years of age [6]. FS can sometimes be unilateral while some individuals may not have this sinus on either side. Since the shape and developmental stages of FS differ in each individual, it can be used to determine the sex and identity of individuals [7]. In 1921, Schuller evaluated the shape, complex structure, and individuality of FS for the first time and stated that it could be used for the identification of individuals in postmortem studies [8].

The aim of this study was to assess the morphological structure of the FS diameters in sex determination using posterioanterior (AP) skull radiography. This data can be valuable for identifying and characterizing human features.

This article was previously presented as a meeting abstract at the 2020 Turkey 21st National Anatomy Congress on November 29, 2020.

## Materials and methods

Patients who underwent AP cranial radiography in our hospital under outpatient conditions were included in the study. A total of 350 patients (171 females, 179 males) aged 20-55 years were included considering that FS continues to develop until the age of 20 and it can expand secondary to resorption after a certain age. Patients with developmental deficiency or pathology, craniofacial syndrome, known endocrine or metabolic disease, and known craniofacial trauma were excluded.

**Figure 1:**
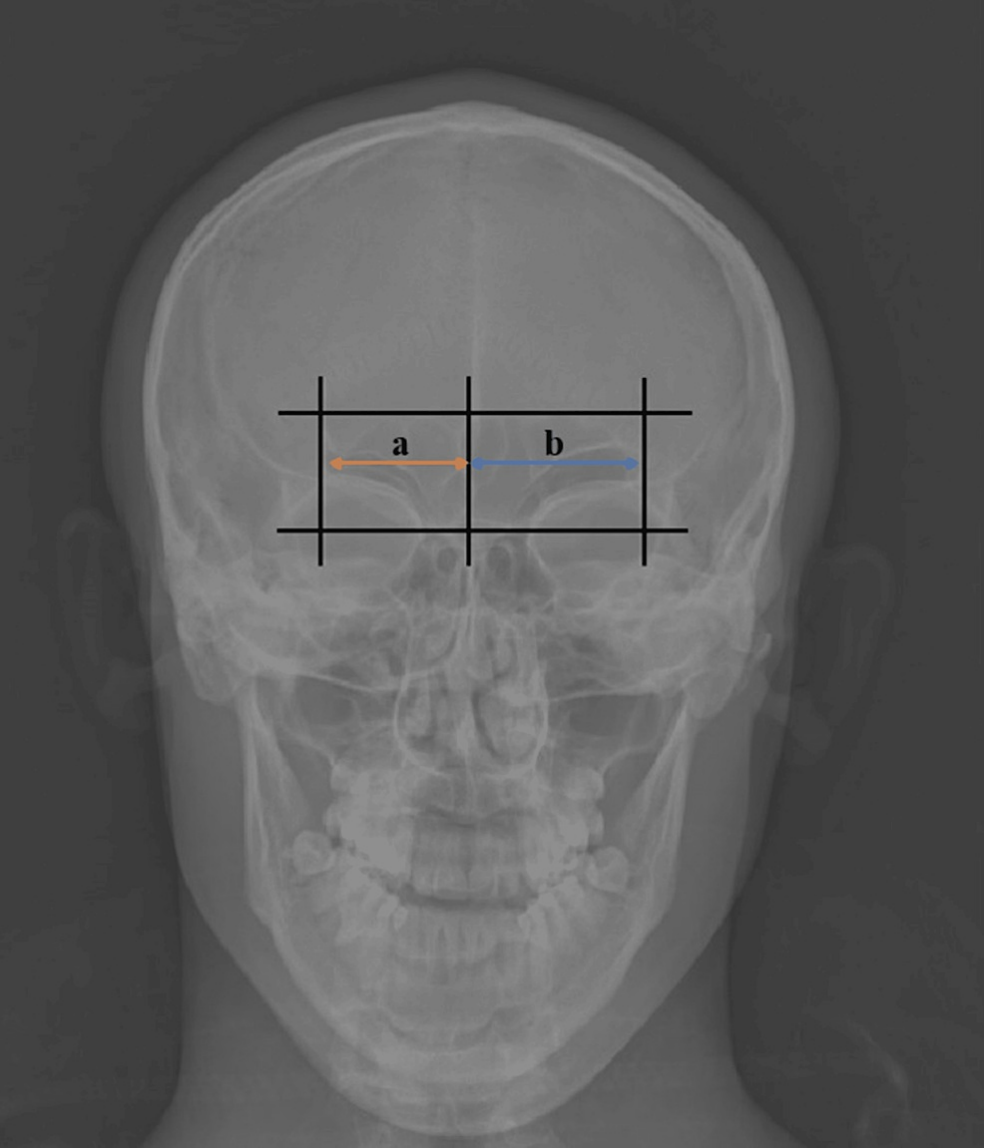
Method used to determine the symmetry and asymmetry of the frontal sinus. If ({a/b} × 100) >20, the frontal sinus was evaluated to be asymmetric. The frame shows the largest diameters of the frontal sinuses. (a) Right frontal sinus width and (b) left frontal sinus width.

FS was anatomically classified as symmetrical, asymmetrical (right or left dominance), and unilateral or bilateral aplasic. The longest dimensions on both sides were divided into each other to evaluate the right and left asymmetry, and the result was multiplied by 100. FS was evaluated as asymmetric if the result was greater than 20% and symmetric if less than 20% (Figure 1) [9,10].

The right and left FS height and width measurements were made based on the largest diameters. For both sides, the width was evaluated as the largest diameter from the central septum, and the height as the upper limit of FS from the frontonasal suture. Patients with bilateral or unilateral aplasia were not included in these measurements.

The Statistical Package for the Social Sciences (SPSS) version 20.0 (Armonk, NY: IBM Corp.) was used for statistical analysis. The independent samples t-test was used to compare the differences in the mean dimensions measured between the males and females. For the use of frontal sinus parameters in sex determination, discriminate analysis, which is one of the multivariate statistical analysis methods, was used to determine whether the units were correctly assigned to their own groups. The linear discriminant function was used for the two groups.

## Results

**Figure 2:**
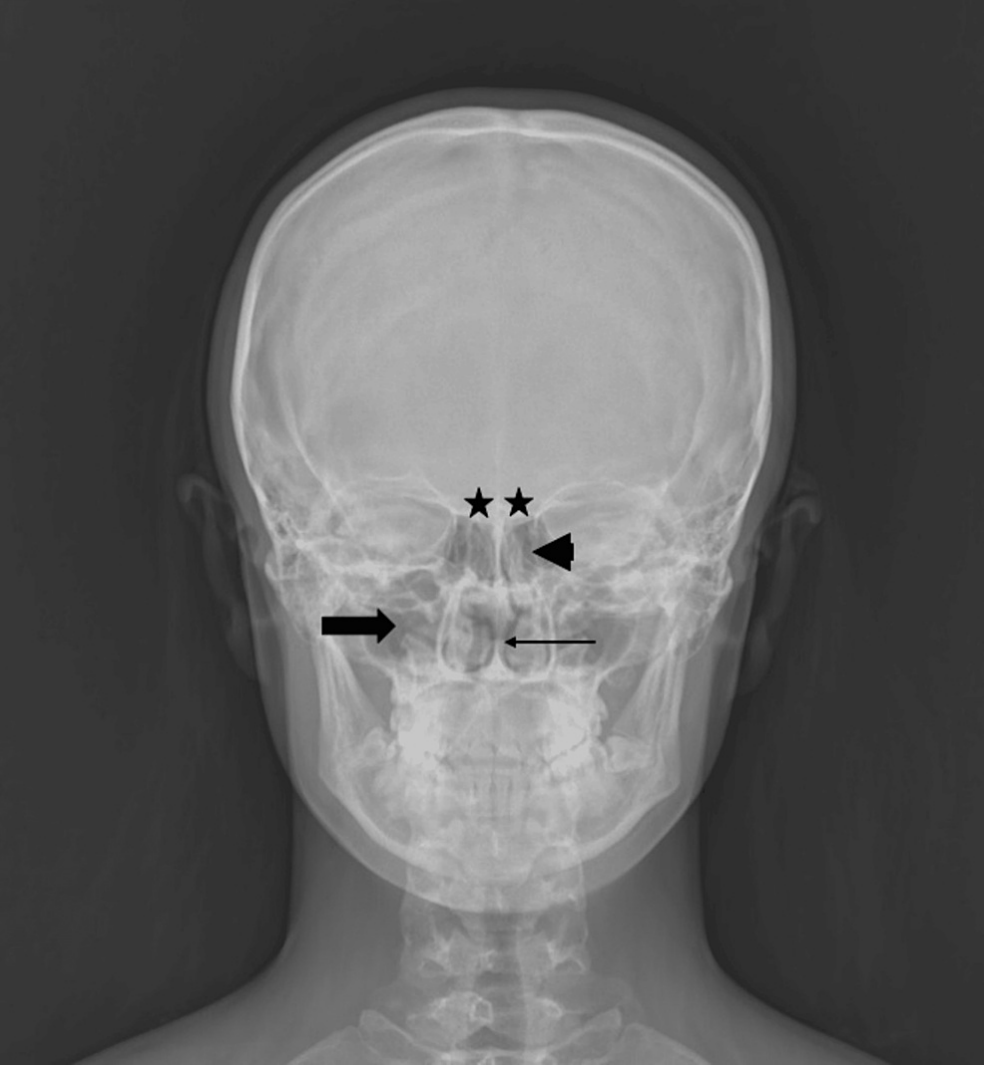
Radiography image of a 20-year-old woman with bilateral frontal aplasia. The image shows bilateral frontal aplasia (star), maxillary sinus (bold arrow), ethmoidal cells (arrowhead), and nasal septum (arrow).

The mean age of all patients included in the study was 32.8±11.45 (20-55) years. The mean age of the males was 31.23±11.9 (20-51) years and that of the females was 34.45±10.75 (20-55) years. When classified anatomically, 20 patients had bilateral aplasia (12 males, eight females), 19 patients had right aplasia (10 males, nine females), and 11 patients had left aplasia (seven males, four females) (Figure 2).

Excluding 50 (14.29%) patients with aplasia, FS was symmetrical in 206 (72.1%) of the remaining 300 patients while right dominance was observed in 33 (9.43%) patients and left dominance in 61 (17.43%) patients. The distribution of findings according to sex is summarized in Table 1. After excluding the patients with aplasia, the measurements were performed in the remaining 300 patients (154 male, 146 female) and the results are presented in Table 2.

**Table 1:** Distribution of frontal sinus patterns.

Classification	Number of individuals		Total (percentage)
	Males	Females	
Symmetry	93 (26.57%)	113 (32.29%)	206 (58.85%)
Left-dominant asymmetry	36 (10.29%)	25 (7.14%)	61 (17.42%)
Right-dominant asymmetry	21 (6%)	12 (3.43%)	33 (9.42%)
Left unilateral aplasia	7 (2%)	4 (1.14%)	11 (3.14%)
Right unilateral aplasia	10 (2.86%)	9 (2.57%)	19 (5.42%)
Bilateral aplasia	12 (3.43%)	8 (2.29%)	20 (5.71 %)

**Table 2:** Descriptive statistics and independent samples t-test parameters studied. SD: standard deviation

Frontal sinus parameters (mm)	Data	Minimum	Maximum	Mean	SD	p-Value (sex)
Right side width	Female	8	47.5	25.52	8.26	<0.001
	Male	18.5	57.8	32.92	7.68	
	Total	8	57.8	29.32	8.78	
Right side height	Female	7.77	50	22.66	8.57	<0.001
	Male	12	64	30.4	10.1	
	Total	7.77	64	26.63	10.14	
Left side width	Female	8.1	51.7	25.79	8.07	<0.001
	Male	11.3	60.1	33.62	8.21	
	Total	8.1	60.1	29.81	9.02	
Left side height	Female	9.37	50.3	23.61	8.77	<0.001
	Male	11.3	61.9	30.93	9.61	
	Total	9.37	61.9	27.37	9.9	

The bilateral mean width values were higher than the height values ​​in both sexes (right p=0.014, left p=0.012 for males; right p=0.004, left p=0.020 for females). The FS height and width values on both sides were all statistically significantly higher in males than in females (p<0.001 for each parameter). However, there was no significant difference between the right and left FS values (width p=0.534, height p=0.676 for males; width p=0.663, height p=0.318 for females).

In the analysis conducted for sex determination, it was determined that the females were accurately classified at a rate of 71.2% and the females at a rate of 68.2%. The accurate classification rate for each sex was calculated as 69.7%.

## Discussion

In the realm of forensic medicine and dentistry, craniometric parameters also play a vital role in both postmortem and antemortem evaluations of individuals. Cranial dimensions can differ among different populations, and measuring the cranium aids in determining racial distinctions and sexes [11]. When we evaluated FS anatomically, we detected the presence of bilateral or unilateral aplasia in 50 patients. FS was symmetrical in 206 of the remaining cases while it was asymmetric in 94 with right or left dominance. We found that the height and width measurements (mm) on both sides were statistically significantly larger in the males compared to the females. All these findings support the thesis that the development of FS is unique and can be used in the identification of individuals, as stated in the literature. In addition, our findings show that FS diameters can also be useful in sex determination.

In our study, we included patients over 20 years of age since it is reported in the literature that FS development can continue up to this age [6]. In addition, we chose to exclude elderly patients from our study considering that after a certain age, FS dimensions are reported to increase secondary to bone resorption [12]. We did not attempt to include an equal number of male and female patients in contrast to some studies in the literature [9,13]. Rather, consistent with some other researchers, we included all patients who met our criteria within a certain time interval [10,14].

Patients with bilateral aplasia constituted 5.71% of our study population. In studies conducted in the literature, this rate varies between 2% and 10% [13-17]. In some studies, no bilateral aplasia was reported [16]. We detected unilateral aplasia in 8.56% of our patients, right aplasia in 5.42% and left aplasia in 3.14%. In the literature, unilateral aplasia has been reported at rates varying between 2.5% and 13.75% [9,13-16]. Our findings are mostly consistent with the previously reported data [11,13-17]. However, the wide range of these rates can be explained by the different genetic, environmental, and ethnic origins of the samples evaluated [18]. For example, a previous study suggested that the absence of FS was very common in the Inuit, which may be a result of adaptation to cold climate [19].

Symmetry was observed in 58.85% of our patients and asymmetry in 26.84% (left dominance in 17.42% and right dominance in 9.42%). In the literature, the FS symmetry and asymmetry have been reported as 55.14-78% and 11.4-23.7%, respectively, which is similar to the values we obtained [9,14,15]. However, there is also a study that found 43.1% symmetrical and 56.6% asymmetrical FS rates, unlike other studies in the literature [20].

In our study, FS diameters on both sides were larger in the males compared to the females. Our results showed that sex determination using FS diameters was successful for 68.2% of the males and 71.2% of the females, while the accuracy rate for both sexes was 69.7%. The literature contains studies using different imaging techniques for sex determination based on FS [21-28]. In a study conducted in India with computed tomography (CT), it was reported that sex determination had an accuracy rate of 61.4% [21], and in a direct radiography study, the accuracy of sex determination was determined as 59% [22]. In the literature using direct skull radiography as in our study, Verma et al. noted that sex determination was undertaken at 61% accuracy [10], while a study conducted in India reported 59.4-64.4% accuracy rates after evaluating the FS diameters on the two sides separately [23], and researchers from Nigeria showed that the accuracy of this method was 60% among patients aged 20-91 years [24].

The highest accuracy rate for sex determination was reported as 80% in a study [4]. However, in contrast to all these studies, there are also a few researchers concluding that FS cannot be used in sex determination. For example, in an Indian study, Goyal et al. found no significant sex difference [17]. Similarly, in a study using conical CT, the accurate sex determination rate was found to be non-significant, being determined as 92% for females and 50% for males [25]. The rates reported in studies conducted in Turkey are similar to those we obtained. Ekizoğlu et al. stated that accurate sex determination was made in 77.5% of females and 70.5% of males. In a study performed with direct skull radiography, it was reported that sex discrimination was successful at a rate of 68.1% [27], while a conical CT study showed a 79% accuracy rate in sex determination [28]. In many studies comparing FS diameters, it has been reported that they are larger in males, which is consistent with our results [23,24,29]. In summary, the frontal sinus is a unique anatomical feature that displays variations in both size and shape across individuals. Our study supports earlier findings, demonstrating that males generally possess larger frontal sinuses than females. These disparities may be linked to distinct developmental patterns and the influence of handedness or footedness, which might potentially affect cerebral dominance [11]. In all these studies, it was found that FS diameters could be used at 59.4-80% accuracy in sex determination. The differences in these percentages may be due to the effects of geographical and environmental factors. It has been reported in the literature that FS diameters have a linear relationship with ambient air temperature and pneumatization. In addition, a relationship between pneumatization and FS diameters and shapes has been reported [19,30].

Our study had certain limitations. First, the number of cases was relatively small. Second, it is known that the morphological characteristics of FS are affected by genetic and environmental factors, and therefore it may not be possible to generalize our data to all populations. Lastly, we were able to evaluate only the parameters of height and width for FS on direct radiography. More parameters can be evaluated with further studies using cross-sectional imaging methods.

## Conclusions

Notably, our research has revealed that frontal sinus diameters are consistently larger in males than in females, and that direct radiography can be employed for gender determination with an accuracy rate of 69.7%. This information underscores the utility of morphometric evaluation of the frontal sinus diameters on direct radiography for gender and identity determination, potentially in conjunction with other parameters. In conclusion, our study has demonstrated the potential of the morphological structure of the frontal sinuses as a valuable tool for identifying and characterizing human individuals. Furthermore, our findings provide clues for medical experts and researchers in the field of craniofacial medicine regarding the complex relationship between frontal sinuses and craniofacial parameters that can be explored in the future, utilizing advanced imaging technology and a specific population focus.
